# Are Individuals Aged 80+ Providing Great‐Grandchild Care More Satisfied With Their Lives and Less Lonely? Findings Based on a Nationwide Representative Sample

**DOI:** 10.1111/psyg.70151

**Published:** 2026-02-20

**Authors:** André Hajek, Supa Pengpid, Karl Peltzer, Hans‐Helmut König

**Affiliations:** ^1^ Department of Health Economics and Health Services Research University Medical Center Hamburg‐Eppendorf, Hamburg Center for Health Economics Hamburg Germany; ^2^ Department of Health Education and Behavioral Sciences, Faculty of Public Health Mahidol University Bangkok Thailand; ^3^ Department of Public Health Sefako Makgatho Health Sciences University Pretoria South Africa; ^4^ Department of Healthcare Administration, College of Medical and Health Science Asia University Taichung Taiwan; ^5^ Department of Psychology University of the Free State Bloemfontein South Africa; ^6^ Department of Medical Research, China Medical University Hospital China Medical University Taichung Taiwan

**Keywords:** aged, 80 and over, great‐grandchildren, happiness, informal care, life satisfaction, loneliness, oldest old, private care, social isolation, successful ageing

## Abstract

**Background:**

Overall, there is a lack of studies examining whether providing great‐grandchild care is associated with life satisfaction and loneliness, based on nationally representative samples. Thus, we aimed to examine whether providing great‐grandchild care is associated with life satisfaction and loneliness (also stratified by sex).

**Methods:**

Data were used from the “Old Age in Germany (D80+)” study—a nationwide representative study encompassing community‐dwelling and institutionalised individuals aged 80+ in Germany (*n* = 995 in the analytic sample). Frequently used tools were used to quantify both loneliness and life satisfaction. Caring for great‐grandchildren served as the key independent variable. It was adjusted for several sociodemographic and health‐related factors. Linear regression models were estimated. Robustness checks were conducted.

**Results:**

Regressions showed that providing great‐grandchild care was not significantly associated with higher life satisfaction among the total sample and men, but among women. Moreover, it was not significantly associated with loneliness (neither in the total sample nor in both sexes).

**Conclusion:**

Providing great‐grandchild care is associated with higher life satisfaction among women aged 80 years and over. Future research in other countries and based on longitudinal data is recommended.

## 1 Introduction

Many parents need grandchild care to balance labour force participation and family obligations, such as supervising their children. Particularly in the light of increasing participation of women in the labour force [1, 2], there is an expected demand for taking care of the children [3]. However, such care is not limited to grandparents alone; great‐grandparents can also sometimes look after their great‐grandchildren. The care provided by great‐grandparents may be somewhat less active than that provided by grandparents. However, the emotional role may be of great importance for the family members. Particularly in light of various critical life events affecting the oldest old individuals (i.e., those aged 80 and above)—such as the death of a partner or friends or health deteriorations—the family, including great‐grandchildren, can be of great value to these individuals. For example, the provision of great‐grandchild care may give life meaning. Additionally, they may have an impact beyond one's own life (e.g., by passing on values to one's great‐grandchildren).

Most of the existing studies, however, have focused on the outcomes of providing grandchild care by grandparents, but not great‐grandparents. Some of these studies showed negative consequences (e.g., in terms of loneliness) of providing grandchild care [4, 5], which may be explained by the role stress theory [6]. Other studies, in contrast, found an association between additional grandchild care (e.g., when the parents are still at work) and a higher likelihood of formal volunteer activity and more social integration [7, 8]. Such findings could be explained by the role enhancement theory [6].

It is worth repeating that the existing studies are predominantly restricted to grandchild care, but not great‐grandchild care. Some studies also did not distinguish between grandchildren and great‐grandchild care (e.g., [9]). Other mainly preliminary studies (based on small, selective samples) focused on the family role and the care situation of great‐grandparents. One of these few studies (*n* = 68, Spain) presented some descriptive findings and bivariate analyses based on individuals with at least one great‐grandchild aged 2 or above [10]. They found that most of the participants considered their role as a great‐grandparent to be a continuation of their role as a grandparent. Another study [11] based on *n* = 103 great‐grandparents in Israel (convenience sample) found that when great‐grandparenting was perceived as purposeful, the motivation for more investment in this role increased. Moreover, frequent contact was observed when married great‐grandmothers lived near their great‐grandchildren [11].

Overall, there is a lack of studies examining whether providing great‐grandchild care is associated with life satisfaction and loneliness, particularly based on nationally representative samples. Therefore, we aimed to investigate whether providing great‐grandchild care is associated with life satisfaction and loneliness using data from a nationally representative sample of individuals aged 80 years and over in Germany (also stratified by sex). Sex‐stratified regressions were conducted because the role of great‐grandmothers (e.g., focusing on everyday care, knowledge transfer and emotional support) may differ from the role of great‐grandfathers (e.g., focusing more on craft skills, spoiling great‐grandchildren and adventure) for their great‐grandchildren (see: [12]). Given the fact that loneliness and life satisfaction are important for successful ageing, morbidity and mortality [13, 14, 15], our present findings are of utmost importance.

## 2 Methods

### 2.1 Sample

For this study, we used data from the ‘Old Age in Germany (D80+)’ study. This study is a nationally representative sample of individuals ≥ 80 years residing in Germany. The D80+ encompasses individuals living in institutionalised surroundings and those living in private households.

The D80+ was conducted by the University of Cologne in collaboration with the Cologne Center for Ethics, Rights, Economics and Social Sciences of Health (ceres) as well as the German Center of Gerontology (DZA). Infas, which is a reputable institute for social and market research, was responsible for collecting the data.

Written questionnaires were used in the D80+ study (with data collection from November 2020 to April 2021). An additional telephone interview was performed from May to October 2021. The written questionnaire included topics of high relevance for the main goals of the D80+ study, such as sociodemographic and health‐related factors. The telephone interviews collected additional information that complemented the study objectives (such as caring for great‐grandchildren) that were included in the telephone interview. It is worth noting that some measures in the interviews required assistance from participants, such as cognitive assessments. Ceres selected which tools would be in the written survey and which in the phone interviews. They aimed to keep the written questionnaire concise to encourage participation among the oldest old. They also expected that response rates would be higher for the written survey compared to the telephone interviews. Further details about the D80+ study are provided elsewhere [16]. The analytic sample consisted of *n* = 995 individuals. It was restricted to individuals having great‐grandchildren (see Section: Key independent variable: Great‐grandchild care).

The D80+ study was ethically approved by the ethical board of the medical faculty (Protocol #: 19‐1387_1). Consent was provided when respondents completed and returned the questionnaire.

### 2.2 Outcome

Satisfaction with life was measured based on a frequently applied single‐item tool. This tool has eleven categories. The endpoints were labelled (0 = ‘completely dissatisfied’; 10 = ‘completely satisfied’). Former research revealed that similar single‐item tools to assess satisfaction outcomes had favourable psychometric characteristics [17].

Loneliness was assessed using a single‐item tool varying from 1 (denoting ‘almost never or never’) to 4 (‘almost always or always’). Similar tools to measure loneliness are frequently used to assess loneliness [15, 18]. Moreover, favourable psychometric properties have been demonstrated [19].

### 2.3 Key Independent Variable: Great‐Grandchild Care

Individuals were asked whether they have regularly or occasionally looked after their great‐grandchildren while their parents were away in the preceding 12 months (response categories: no or yes). Gainful employment and voluntary work (e.g., as nursery school teacher or kindergarten teacher) were not included.

This question was only asked to those who actually have great‐grandchildren. Thus, worth repeating, this study only compares individuals who have great‐grandchildren but do not care for them with individuals who have great‐grandchildren and do care for them.

### 2.4 Covariates

Guided by previous research [20, 21, 22, 23] and based on theoretical considerations, covariates were chosen. For example, age may be associated with great‐grandchild care and also with loneliness and life satisfaction [24]. Another example: Functional impairment might also be linked with great‐grandchild care and the outcomes used [25].

Concerning sociodemographic factors, it was adjusted for age, sex (men or women), marital status (married, living together with spouse; married, living separated from spouse; divorced; widowed; single) and education (according to the ISCED‐11 classification [26]: low education; medium education; high education). Health‐related covariates encompassed self‐rated health (single‐item tool, from 1 = very bad to 4 = very good), a count of chronic conditions (based on 22 chronic conditions such as cancer, mental illness, stroke or heart attack; the chronic conditions are based on the multimorbidity index in old age [27]), and functional impairment. Based on seven items, a modified version of the Instrumental Activities of Daily Living (IADL) tool developed by Lawton et al. [28] was used. A final score was calculated by averaging all seven items. The final score varies from 0 to 2, whereby higher values denote higher functional abilities. In a sensitivity analysis, functional impairment was replaced by cognitive impairment. It was measured by using the DemTect screening tool [29, 30], with total scores varying from 0 to 18 (higher scores reflect less cognitive impairment). Scores varying from 9 to 12 reflect mild cognitive impairment and scores lower than 9 reflect dementia. Scores from 13 to 18 reflect the absence of cognitive impairment.

### 2.5 Statistical Analysis

As a first step, the characteristics of the analytic sample (also stratified by providing great‐grandchild care) are depicted. Afterwards, unadjusted and adjusted linear regressions (with cluster‐robust standard errors) were employed to examine the association of great‐grandchild care with loneliness and life satisfaction. It should be noted that except for the variables relating to caring for great‐grandchildren and cognitive impairments (both of which were collected during the telephone interview), all other variables used in this study were collected as part of the written questionnaire.

Missing data were addressed using a full‐information maximum likelihood (FIML) approach. In a sensitivity analysis, missing data were handled using listwise deletion. In such model, a HC3 bias correction (jackknife estimator) with Hansen adjustments [31] was calculated for the standard errors.

We also performed a sensitivity analysis with ordered logistic regressions (rather than linear regressions) when loneliness served as the outcome measure.

Regarding multicollinearity, we calculated variance inflation factors (VIFs) across the six regressions. The highest mean VIF was 1.25 (highest VIF was 1.58 in this model for IADL). Thus, one can conclude that multicollinearity is not a threat.

Statistical significance was determined at *p* < 0.05 in our present study. Statistical analysis was performed using StataNow 19.5 MP‐Parallel Edition (Stata Corp., College Station, Texas).

## 3 Results

### 3.1 Sample Characteristics

Sample characteristics for the weighted analytic sample (*n* = 995 individuals) are shown in Table 1. In total, the average age was 86.9 years (SD: 4.5; 80 to 100 years), and 55.4% of the respondents were female. Stratified by providing great‐grandchild care (no or yes), the average life satisfaction score was 7.4 (SD: 2.0) among individuals not providing great‐grandchild care, whereas it was 7.9 (SD: 1.7) among individuals providing great‐grandchild care. While the average loneliness score was 1.6 (SD: 0.7) among individuals not providing great‐grandchild care, it was 1.5 (SD: 0.7) among individuals providing great‐grandchild care. Further details are presented in Table 1.

**TABLE 1:** Sample characteristics (analytic sample), also stratified by providing great‐grandchild care.

Variables	Providing great‐grandchild care		Total sample	*p*
	No 930 (93.5)	Yes 65 (6.5)	995 (100.0)	
	*N* (%)/Mean (SD)	*N* (%)/Mean (SD)	*N* (%)/Mean (SD)	
Sex				0.44
Men	412 (44.3)	32 (49.2)	444 (44.6)	
Women	518 (55.7)	33 (50.8)	551 (55.4)	
Age (in years)	86.9 (4.5)	86.0 (4.5)	86.9 (4.5)	0.10
Marital status				0.01
Married, living together with spouse	379 (41.4)	37 (57.8)	416 (42.4)	
Married, living separated from spouse	7 (0.8)	2 (3.1)	9 (0.9)	
Divorced	30 (3.3)	3 (4.7)	33 (3.4)	
Widowed	493 (53.8)	22 (34.4)	515 (52.6)	
Single	7 (0.8)	0 (0.0)	7 (0.7)	
Education				0.04
Low education	178 (19.8)	6 (9.8)	184 (19.1)	
Medium education	457 (50.7)	29 (47.5)	486 (50.5)	
High education	266 (29.5)	26 (42.6)	292 (30.4)	
Count of chronic conditions	5.2 (3.1)	5.3 (3.6)	5.2 (3.1)	0.74
Self‐rated health (1 to 4; higher values reflect a more favourable self‐rated health)	2.7 (0.7)	2.8 (0.6)	2.7 (0.7)	0.18
Functional impairment (0 to 2; higher values reflect a higher functional ability)	1.4 (0.6)	1.6 (0.5)	1.5 (0.6)	0.02
Life satisfaction (from 0 to 10; higher values reflect a higher life satisfaction)	7.4 (2.0)	7.9 (1.7)	7.5 (2.0)	0.05
Loneliness (1 to 4, higher values reflect higher loneliness levels)	1.6 (0.7)	1.5 (0.7)	1.6 (0.7)	0.12

*Note:* *p* values are based on Chi^2^‐tests or one‐way ANOVAs, as appropriate.

### 3.2 Regression Analysis

In Table 2, findings of linear regressions are displayed (worth repeating: FIML was used to address missing data). Unadjusted findings are presented in Table S1. Providing great‐grandchild care was not significantly associated with life satisfaction among the total sample and men, but it was among women (*β* = 0.68, *p* < 0.01). Providing great‐grandchild care was not significantly associated with loneliness in the total sample or in either sex. In a robustness check, listwise deletion was used to handle missing data. The findings remained nearly the same: only providing great‐grandchild care was significantly associated with life satisfaction among women (*β* = 0.64, *p* = 0.03). It may also be worth noting that this association persisted even when adjusted for the living situation (i.e., community‐dwelling vs. living in institutionalised settings). When ordered logistic regressions were used rather than linear regressions (with loneliness as the outcome measure), the findings remained virtually the same in terms of significance (please see Table S2 for more details). In a further sensitivity analysis, the covariate functional impairment was replaced by cognitive impairment. However, the findings remained nearly identical for the association of great‐grandchild care and the outcomes used (please see Table S3 for additional details).

**TABLE 2:** Association of great‐grandchild care with loneliness and life satisfaction: Results of multiple linear regressions.

Independent variables	Life satisfaction among the total sample	Life satisfaction among men	Life satisfaction among women	Loneliness among the total sample	Loneliness among men	Loneliness among women
Providing great‐grandchild care: yes (reference category: no)	0.31	−0.06	0.68**	−0.04	−0.03	−0.02
	(−0.06 to 0.69)	(−0.60 to 0.49)	(0.17 to 1.19)	(−0.20 to 0.13)	(−0.23 to 0.17)	(−0.27 to 0.24)
Covariates	✓	✓	✓	✓	✓	✓
Observations	995	444	551	998	440	558
*R* ^2^	0.17	0.15	0.19	0.13	0.19	0.08

*Note:* Unstandardized beta‐coefficients are displayed; 95% CI in parentheses; ***p < 0.001, **p < 0.01, *p < 0.05, +*p* < 0.10; Covariates include: sex (if applicable), age, marital status, education, self‐rated health, functional impairment, and a count of chronic conditions.

## 4 Discussion

Using data from a nationally representative sample of individuals aged 80 years and over in Germany, we aimed to investigate whether providing great‐grandchild care is associated with life satisfaction and loneliness (also stratified by sex). Our key findings were as follows: Providing great‐grandchild care was not significantly associated with life satisfaction among the total sample and men, but it was among women (*β* = 0.68, *p* < 0.01). Providing great‐grandchild care was not significantly associated with loneliness in the total sample or in either sex. Our present nationally representative study adds to our current understanding of great‐grandchild care and psychosocial outcomes, mainly based on very few studies based on small, selective samples (e.g., [10, 11]). Due to the lack of similar studies (particularly explicitly referring to great‐grandchild care), it is difficult to compare our present findings with previous research. More broadly, previous research concerning grandchild care and psychosocial outcomes mainly showed somewhat mixed evidence [4, 5, 6, 32].

While great‐grandchild care was not significantly associated with satisfaction with life among men, it was associated with higher life satisfaction among women. Such findings are in accordance with the role enhancement theory. We believe that great‐grandchild care may give life meaning among women in particular [10]. They may feel valued and needed (see: [11]). For example, if the parents have important work commitments later in the day, and the great‐grandchild urgently needs to be looked after in the afternoon, or when the great‐grandchild is sick and supervision is immediately required. It could also give them pleasure and a meaning in life to impart values and knowledge to have a lasting (beyond death) impact (see: [12]). The emotional support of their great‐grandchildren could also bring them joy. Our findings may also stress the fact that family‐related factors are of particularly high relevance among women [33, 34].

Moreover, differences in the type and duration of great‐grandchild care between the sexes could also explain the results. For example, women may be more often involved in practical support (such as feeding or comforting) and emotional care, whereas men may be more involved in playful activities (see also: [12]). In line with theories of gender norms in family roles [35], women are probably more involved in caring for their great‐grandchildren than men, which in turn could be related to life satisfaction.

Interestingly, the provision of great‐grandchild care was not associated with loneliness in our study. It may be the case that other factors, such as spousal loss—with whom individuals may have had a relationship lasting over many stages of life—may be of greater importance for unmet social needs among the oldest old, as highlighted by a recent systematic review [15].

Some strengths and limitations should be borne in mind when interpreting our current results. Data came from a nationwide sample of individuals aged 80 years and over in Germany. The sample encompassed both individuals living in institutionalised settings and those residing in private households. Several robustness checks were conducted. Single‐item tools with high face validity were used to quantify loneliness and life satisfaction. Nevertheless, we recommend future research based on multi‐item tools, for example, to distinguish between emotional and social loneliness. Due to reasons of data availability, our study focused on general great‐grandchild care. More details on great‐grandchild care are not provided in the D80+ study. Future research is therefore suggested to investigate further topics such as the exact amount of time providing care, the type of activity (e.g., transportation, supervision, playing or feeding), the characteristics of the great‐grandchildren (e.g., age, health status) or the relationship quality between great‐grandchildren and great‐grandparents. The cross‐sectional design is a further shortcoming of this study. Moreover, our findings may be affected by the time of data collection (i.e., during the pandemic). The data on great‐grandchild care was collected during a period when restrictions were significantly relaxed in Germany. Nevertheless, it can be assumed that the intergenerational contact was probably significantly different (e.g., in terms of the type and length of great‐grandchild care) than before or after the pandemic. In this respect, it is somewhat questionable to what extent our findings can be generalised to other periods. Thus, future research in the post‐pandemic era is recommended.

Furthermore, due to data availability, it was not possible to adjust for childcare responsibilities for grandchildren. It should also be noted that the outcomes were collected in the written questionnaire (worth repeating: November 2020 to April 2021) and the question about great‐grandchild care in the telephone interview (worth repeating: May to October 2021). In this respect, the possibility of reverse causality in particular cannot be dismissed. Therefore, future research (e.g., based on multicentre prospective cohort studies) is recommended. Furthermore, it should be noted that written questionnaires/telephone interviews were conducted (rather than face‐to‐face interviews) due to the Covid‐19 pandemic.

In conclusion, providing great‐grandchild care is associated with higher life satisfaction among women aged 80 years and over in Germany. Upcoming studies using longitudinal data from other countries are needed.

## Funding

The authors have nothing to report.

## Disclosure

The authors have nothing to report.

## Ethics Statement

The ethical board of the medical faculty at the University of Cologne (Protocol #: 19‐1387_1) approved the D80+ study. The interviews were only conducted with the consent of the interviewees. The questionnaire itself contains a brief introduction and the privacy policy. Consent is given when the respondents complete and return the questionnaire.

## Consent

The authors have nothing to report.

## Conflicts of Interest

The authors declare no conflicts of interest.

## Supporting information


**Table S1:** Association of great‐grandchild care with loneliness and life satisfaction: results of unadjusted linear regressions.
**Table S2:** Association of great‐grandchild care with loneliness: results of ordered logistic regressions.
**Table S3:** Association of great‐grandchild care with loneliness and life satisfaction: results of multiple linear regressions (with cognitive impairment rather than functional impairment as covariate).

## Data Availability

All data are available from the German Centre of Gerontology. For further details (application for data use): https://www.dza.de/en/research/fdz/access‐to‐data/application.
