# Supplementary material for: Are Individuals Aged 80+ Providing Great‐Grandchild Care More Satisfied With Their Lives and Less Lonely? Findings Based on a Nationwide Representative Sample
Source: Psychogeriatrics. 2026 Feb 20;26(2):e70151. doi: 10.1111/psyg.70151 (PMC12923383; doi:10.1111/psyg.70151)
Supplement: Supplementary file 1 — Table S1: Association of great‐grandchild care with loneliness and life satisfaction: results of unadjusted linear regressions. Table S2: Association of great‐grandchild care with loneliness: results of ordered logistic regressions. Table S3: Association of great‐grandchild care with loneliness and life satisfaction: results of multiple linear regressions (with cognitive impairment rather than functional impairment as covariate). [file PSYG-26-0-s001.docx]

Supplementary Table 1. Association of great-grandchild care with loneliness and life satisfaction: results of unadjusted linear regressions.

| Independent variables | Life satisfaction among the total sample | Life satisfaction among men | Life satisfaction among women | Loneliness among the total sample | Loneliness among men | Loneliness among women |
| --- | --- | --- | --- | --- | --- | --- |
|  |  |  |  |  |  |  |
| Providing great-grandchild care: yes (reference category: no) | 0.49* | -0.01 | 0.94** | -0.14+ | -0.15 | -0.11 |
|  | (0.07 - 0.92) | (-0.64 - 0.61) | (0.38 - 1.51) | (-0.31 - 0.03) | (-0.37 - 0.06) | (-0.36 - 0.14) |
|  |  |  |  |  |  |  |
| Observations | 1,013 | 453 | 560 | 1,016 | 449 | 567 |
| R² | 0.004 | 0.000 | 0.01 | 0.002 | 0.004 | 0.001 |

Notes: Unstandardized beta-coefficients are displayed; 95% CI in parentheses; *** p<0.001, ** p<0.01, * p<0.05, + p<0.10;

Supplementary Table 2. Association of great-grandchild care with loneliness: results of ordered logistic regressions.

| Independent variables | Loneliness among the total sample | Loneliness among men | Loneliness among women |
| --- | --- | --- | --- |
|  |  |  |  |
| Providing great-grandchild care: yes (reference category: no) | 0.91 | 1.04 | 0.91 |
|  | (0.51 - 1.61) | (0.44 - 2.42) | (0.41 - 2.02) |
|  |  |  |  |
| Covariates | ✓ | ✓ | ✓ |
| Observations | 931 | 417 | 514 |
| Pseudo R² | 0.07 | 0.11 | 0.04 |

Notes: Odds Ratios are displayed; 95% CI in parentheses; *** p<0.001, ** p<0.01, * p<0.05, + p<0.10; Covariates include: sex (if applicable), age, marital status, education, self-rated health, functional impairment, and a count of chronic conditions.

Supplementary Table 3. Association of great-grandchild care with loneliness and life satisfaction: results of multiple linear regressions (with cognitive impairment rather than functional impairment as covariate).

| Independent variables | Life satisfaction among the total sample | Life satisfaction among men | Life satisfaction among women | Loneliness among the total sample | Loneliness among men | Loneliness among women |
| --- | --- | --- | --- | --- | --- | --- |
|  |  |  |  |  |  |  |
| Providing great-grandchild care: yes (reference category: no) | 0.32+ | -0.05 | 0.71* | -0.03 | -0.03 | -0.01 |
|  | (-0.06 - 0.71) | (-0.58 - 0.48) | (0.17 - 1.26) | (-0.20 - 0.13) | (-0.22 - 0.17) | (-0.27 - 0.25) |
|  |  |  |  |  |  |  |
| Covariates | ✓ | ✓ | ✓ | ✓ | ✓ | ✓ |
|  |  |  |  |  |  |  |
| Observations | 995 | 444 | 551 | 998 | 440 | 558 |
| R² | 0.16 | 0.14 | 0.18 | 0.13 | 0.18 | 0.08 |

Notes: Unstandardized beta-coefficients are displayed; 95% CI in parentheses; *** p<0.001, ** p<0.01, * p<0.05, + p<0.10; Covariates include: sex (if applicable), age, marital status, education, self-rated health, cognitive impairment, and a count of chronic conditions.
